# *Notes from the Field:* Reference Laboratory Investigation of Patients with Clinically Diagnosed Lyme Disease and Babesiosis — Indiana, 2016

**DOI:** 10.15585/mmwr.mm6741a6

**Published:** 2018-10-19

**Authors:** Jennifer A. Brown, Rex Allman, Barbara L. Herwaldt, Elizabeth Gray, Hilda N. Rivera, Yvonne Qvarnstrom, Natalie Kwit, Martin E. Schriefer, Alison Hinckley, Pamela Pontones

**Affiliations:** ^1^Indiana State Department of Health; ^2^Pulaski County Health Department, Winamac, Indiana; ^3^Division of Parasitic Diseases and Malaria, Center for Global Health, CDC; ^4^Division of Vector-Borne Diseases, National Center for Emerging and Zoonotic Infectious Diseases, CDC.

In the midwestern United States, the principal vector for Lyme disease (*Borrelia burgdorferi*) and babesiosis (*Babesia microti*) is the *Ixodes scapularis* tick, which has been documented in 77 of 92 Indiana counties (Indiana State Department of Health [ISDH], unpublished data, 2018) (*1*). The average annual Lyme disease incidence in Indiana is low (1.3 cases per 100,000 population during 2011–2015) (*2*); however, rates in some northwestern counties are higher (*3*). A two-tiered serologic testing algorithm is recommended for diagnosing Lyme disease (*4*). Babesiosis is rare in Indiana, with no confirmed cases and one probable case reported during 2011–2015. Blood smear examination or polymerase chain reaction (PCR) analysis are typically recommended for the diagnosis of acute babesiosis (*5*). In June 2016, a physician in northwestern Indiana informed ISDH of a high prevalence of clinically diagnosed Lyme disease among his patients. He further reported that eight patients evaluated during 2015–2016 had tested positive for *B. microti *immunoglobulin G (IgG) or immunoglobulin M (IgM) antibodies by enzyme immunoassay (EIA) at a commercial laboratory. To further evaluate these findings, ISDH and CDC conducted a laboratory investigation using specimens from some of the patients.

The physician in northwestern Indiana was asked to select clinically representative patients for further investigation; 14 were chosen, including five of the eight who had positive *B. microti* EIA results (Table). Whole blood and serum specimens were collected and tested at CDC for evidence of *Borrelia* and *Babesia* infection. ISDH did not conduct patient interviews or chart reviews; demographic and clinical data were obtained from the CDC specimen submission form. Clinical manifestations reported in an unstructured memo field were compared with national surveillance case definition clinical criteria for Lyme disease and babesiosis (*6*). CDC tested for Lyme disease by whole cell sonicate and C6 peptide EIAs followed by IgM and IgG immunoblots for all patients and for *Babesia* infection by examination of Giemsa-stained blood smears, PCR, and indirect fluorescent antibody (IFA) for total immunoglobulin to *B. microti*.

**TABLE:** Demographic data, illness onset dates, selected clinical manifestations, and selected laboratory results for 14 patients with clinically diagnosed Lyme disease and babesiosis — Indiana, 2016

Patient	Age (yrs)	Sex	Onset date	Clinical manifestation		*Babesia microti* serology (commercial laboratory EIA)		CDC results			
				Objective	Subjective	IgG	IgM	Lyme disease serology			Total Ig titer to *B*. *microti* (IFA)*
								WCS EIA	C6 peptide EIA	IgM/IgG immunoblot	
A	57	F	09/2015	None	Sweats, headache, myalgia	pos	neg	neg	neg	neg	<1:8
B	50	F	09/2015	Rash^†^	Sweats, headache, myalgia	pos	neg	neg	neg	neg	<1:8
C	31	F	11/2015	None	Headache, myalgia	pos	neg	neg	neg	neg	<1:8
D	46	M	06/2016	None	Sweats	pos	neg	neg	neg	neg	<1:8
E	68	F	08/2015	Anemia^†^	Sweats, headache, myalgia	neg	pos	neg	neg	neg	<1:8
F	51	F	07/2015	Fever^†^	Headache, myalgia, arthralgia	^—§^	^—§^	neg	neg	neg	1:64
G	50	M	06/2016	None	Sweats, myalgia, arthralgia	^—^	^—^	neg	neg	neg	1:64
H	76	F	07/2015	None	Sweats, myalgia, arthralgia	^—^	^—^	neg	neg	neg	<1:8
I	31	M	01/2016	None	Sweats, myalgia, arthralgia	^—^	^—^	neg	neg	neg	<1:8
J	40	F	01/2016	None	Myalgia, arthralgia	^—^	^—^	neg	neg	neg	<1:8
K	43	F	01/2016	Rash^†^	Headache, myalgia, arthralgia	^—^	^—^	neg	neg	neg	<1:8
L	30	F	01/2016	NP	NP	^—^	^—^	neg	neg	neg	<1:8
M	45	M	03/2016	None	Sweats, myalgia	^—^	^—^	neg	pos	neg	<1:8
N	10	M	04/2016	Rash^†^	Myalgia	^—^	^—^	neg	neg	neg	<1:8

**Abbreviations**: EIA = enzyme immunoassay; F = female; IFA =  indirect fluorescent antibody; Ig = immunoglobulin; IgG = immunoglobulin G; IgM = immunoglobulin M; M = male; neg = negative; NP = not provided; pos = positive; WCS = whole cell sonicate.

* For patients A–E, the specimens tested by IFA were collected a median of 282 days (range = 30–323 days) after the specimens tested by EIA.

^†^ Details not specified.

^§^ Not done.

The 14 patients lived in seven northwestern Indiana counties. The median age was 46 years (range = 10–76 years); nine were female (Table). The only reported objective clinical manifestations potentially consistent with Lyme disease were unspecified rashes in three patients (B, K, and N). Objective manifestations consistent with babesiosis included anemia (patient E) and fever (patient F). A median of three prescribed antimicrobial agents (range = 1–6) were reported per patient, without mention of indications. Exposure and travel histories were not provided.

Patient specimens were collected a median of 172 days (range = 22–348 days) after reported illness onset dates; the interval was ≥3 months for all but two patients (D and G). One patient (M) had positive C6 peptide EIA results; no patient had positive whole cell sonicate EIA or immunoblot results (Table). All patient serologies were therefore interpreted to be negative for Lyme disease (*4*). Two patients (F and G) had *B. microti* IFA titers of 1:64; they reportedly became symptomatic in July 2015 and June 2016, respectively. The results of all other *Babesia* testing were negative.

This laboratory-based investigation does not suggest a cluster of Lyme disease or babesiosis cases among these patients. None had serologic evidence of Lyme disease or parasitologic or molecular evidence of *Babesia* infection, and only two had serologic evidence of *B. microti* infection. A *B. microti* IFA titer of 1:64 is insufficient laboratory evidence to fulfill the national surveillance case definition for non–transfusion-associated babesiosis (*6*) and could reflect early, chronic, or resolved infection or nonspecific reactivity.

Lyme disease and babesiosis should be considered in the differential diagnosis for patients with clinically compatible illness and potential exposure to *I. scapularis* ticks in areas where the pathogens are present. Physicians in low-prevalence states can increase the positive predictive value of laboratory testing by carefully selecting patients for testing, following established diagnostic recommendations, and using certified or accredited laboratories (*5*,*7*).
